# Between and within-person relations between psychological wellbeing and distress in adolescence: A random intercept cross-lagged panel examination

**DOI:** 10.1017/S0954579425100187

**Published:** 2025-05-28

**Authors:** Hena Thakur, Jae Wan Choi, Jeff R. Temple, Joseph R. Cohen

**Affiliations:** 1 Department of Medical Social Sciences, Northwestern University Feinberg School of Medicine, Chicago, IL, USA; 2 Department of Psychology, University of Illinois at Urbana-Champaign, Urbana, IL, USA; 3 School of Behavioral Health Sciences, UTHealth, Houston, TX, USA

**Keywords:** adolescence, holistic mental health, longitudinal

## Abstract

Holistic frameworks of mental health outline that a focus on psychopathology does not represent an optimal approach to defining, measuring and treating mental health. Rather, theoretical, empirical, and applied psychological efforts should incorporate psychological well-being (PWB). Studies of PWB have overwhelmingly focused on adult populations, rendering a translation down to adolescence difficult. The current study explores the between-person, as well as within-person short-term, prospective relations between psychopathology and wellbeing within a community sample of adolescents (i.e., 553 youth aged 12 – 18, mean age: 14.97 years, 51.2% Male, 40.7% of participants identified as Hispanic (225 individuals), 38.5% identified as White (213 individuals), and 35.6% identified as Black (197 individuals), 3-wave, 1-year survey). Results demonstrated significant, negative between-person relations between psychopathology and PWB (*b*_PHQ_ = −0.25, SE = 0.11, *p* = 0.021, *b*_VDS_ = −0.39, SE = 0.15, *p* = 0.011). At the within-person level, consistent positive prospective relations were identified for violent-delinquent behaviors and PWB, such that increases in individual levels of violent-delinquent behaviors tended to forecast higher levels of PWB at the next follow-up (*b*_PWBW2_ = 0.21, SE_PWBW2_ = 0.076, *p* < 0.01; *b*_PWBW3_ = 0.14, SE_PWBW3_ = 0.051, *p* < 0.01). At the within-person level, prospective relations between depressive and PWB were not identified. Gender and racial/ethnic identities did not moderate findings.

## Introduction

Holistic frameworks of mental health theorize that positive states of being do not occur in the absence of psychopathology, but rather simultaneously (e.g., Keyes et al., 2010). Translationally, these frameworks posit that decreasing or ameliorating distress symptoms is a suboptimal threshold within the field of mental health, and instead, efforts should focus on conceptualizing and promoting psychological well-being as well (e.g., Howell et al., 2013). To date, however, a holistic framework has not been comprehensively empirically explored, which inhibits its translation into mental health practice. Given increasing interest in clinical initiatives that utilize holistic mental health treatment within adolescence (e.g., Ruini et al., 2009), our study focused on the prospective relation between psychological well-being (PWB) and subtypes of psychopathology. Specifically, given the personal and societal costs associated with adolescent depression (combination of some or all of the following symptoms: sadness, anhedonia, changes in appetite, fatigue, psychomotor agitation or retardation, sleep difficulties, worthlessness or guilt, concentration difficulties, persistent thoughts of death; Petito et al., 2020) and violent-delinquent behaviors (Cohen & Piquero, 2009; e.g., tendency to use physical force in interpersonal interactions, nonphysical aggression; Broidy et al., 2003), we examined how PWB may influence, or be influenced, by these two outcomes.

Psychological wellbeing (PWB) reflects personal fulfillment and the realization of one’s potential (Keyes, 2007), which, in turn, fosters goals and meaning in life (Ryan & Deci, 2001). Ryff’s measure of PWB (1989) is an important index of eudaimonic wellbeing and conceptualization of this construct consists of several facets, including self-acceptance (holding a positive view of oneself even with awareness of one’s limitations), positive relations with others (developing and maintaining close and trusting relationships with others), environmental mastery (shaping one’s environment to meet needs), autonomy (evaluating oneself by their own standards/resistance to societal pressures to behave or think a certain way), purpose in life (meaning making in challenges, having goals and a sense of direction in life), and personal growth (capitalizing on personal strengths). As such, PWB represents tasks that individuals navigate when realizing their potential, capacity or strengths (Ryff et al., 2021). Previous literature examining the psychometric properties of PWB (e.g., Gao & McLellan, 2018; Thakur et al., 2024) provides support for the reliable and valid measurement of this construct within adolescence. Despite growing empirical interest in this index, few studies examine the bidirectional relation between PWB and psychopathology, precluding an understanding of its role in mental health. At the cross-sectional level, correlational studies demonstrate small to medium negative associations between psychopathology and PWB (depressive symptoms: Ford et al., 2013; depressive symptoms and externalizing behaviors; Inguglia et al., 2015), suggesting the two relate to each other. Among adults, there is a significant bidirectional relation between distress and positive well-being (which combines PWB and hedonic well-being); however, changes in psychopathology better account for variance in future positive mental health outcomes than vice versa (Lamers et al., 2015). To date, it is unclear though how PWB may relate to distress in adolescents. Recent research demonstrates that PWB may reflect a single factor in adolescents comprised of Ryff’s original domains (Thakur et al., 2024) as opposed to the multifaceted construct in adulthood (Ryff & Keyes, 1995). This suggests that PWB may present differently in adolescents and have distinct relations to other forms of well-being. Thus, given these developmental differences, separating PWB from hedonic well-being and examining its prospective relation to psychopathology within adolescence, is important for developing holistic models of mental health that are developmentally appropriate.

A secondary aim of the present study sought to parse between and within-person relations between positive and negative forms of mental health. Separating between and within-person effects is not only important for statistical conceptualizations of psychological phenomenon (Hamaker et al., 2015) but also has significant translational implications. Specifically, between-person effects can help inform population mental health efforts (e.g., screening), while the effects at the individual level can provide insight into the cascading effects of intervening with a given psychological construct. To date, Kraiss and colleagues (2022) represents one of the few empirical investigations exploring between- and within-person associations between internalizing distress (i.e., anxiety and depressive symptoms) and wellbeing (i.e., PWB and hedonic wellbeing). In a sample of 25 university students, the authors conducted an intensive longitudinal design study and, using a multilevel modeling framework, found a negative relation between psychological distress and wellbeing at the between and within-levels. In addition, Joshanloo & Blasco-Belled (2023) used a random-intercept cross lagged panel approach to understand the relations between depressive symptoms, eudaimonic wellbeing and life satisfaction in a sample of 17,056 participants with a mean age of 58.8 years at the first timepoint. The authors found that all cross-lagged effects were significant, with life satisfaction and eudaimonic wellbeing demonstrating positive effects and depressive symptoms demonstrating negative effects with life satisfaction and eudaimonic wellbeing. These findings, when combined with Lamers and colleagues (2015), suggest that, if replicated in a larger, more representative sample of adolescents across a longer duration of time, reducing psychological distress across the lifespan may be beneficial for increasing PWB.

Importantly, the above literature limited their studies to examining linear combinations of positive and negative mental health states, despite potential for the relations to be non-linear. For instance, although emotional clarity is positively and linearly associated with PWB (Augusto-Landa et al., 2011), others have demonstrated that higher *and* lower levels of emotional clarity are associated with increased internalizing distress (Park & Naragon-Gainey, 2019). This suggests that there may be processes that influence the relation between internalizing distress and PWB, such that there are comparable, nonlinear presentations of distress across the spectrum of PWB profiles. Considering curvilinear relations may also be important for externalizing (e.g., violent or rule-breaking behaviors) forms of psychopathology. Within adolescence, risk taking can be adaptive and socially reinforced until a certain limit, as it is associated with a variety of functional outcomes that may pave the way toward higher PWB (e.g., pursuit of goals; Sijtsema et al., 2020). Further, Panayiotou and Humphrey (2018) found that externalizing problems in adolescence contribute to reduced emotional distress in the future. The authors hypothesized this may be due to social support from delinquent peers leading to higher self-esteem at this developmental stage, as well as increased attention from adults, which can serve as a protective factor in the aftermath of future stressors. Thus, in line with broader literature examining mental health as a non-linear phenomenon (e.g., Seery et al., 2010), it may be that externalizing symptoms demonstrate a quadratic existence with wellbeing, such that lower and higher levels of externalizing symptoms correspond to lower states of PWB. As such, another aim of the current study was to examine the linearity of the relations between PWB and psychopathology.

Finally, given the established role of adolescent identity in distress outcomes (e.g., Nolen-Hoeksema & Girgus, 1994), it is important to consider how the relation between psychopathology and PWB varies across subpopulations. For example, females are more likely to ruminate, which may sometimes lead towards positive outcomes (e.g., reflection), but also may engage in repetitive thought processes that lead to emotional distress (e.g., brooding; Burwell & Shirk, 2007). Therefore, it is possible that a closer relation between internalizing distress and PWB exists for girls relative to boys. For externalizing symptoms findings are mixed, with some showing that overt forms of aggression are associated with adaptive functioning among adolescent boys (e.g., Warren et al., 2016), other studies showing that externalizing behaviors are equivalently positive across genders (e.g., popularity; Prinstein & Cillessen, 2003), and other research suggesting more negative consequences for rule-breaking in boys relative to girls (Liu et al., 2023). Thus, explicit research on how PWB and externalizing behaviors may vary across genders is necessary. Meanwhile, the relation between PWB and psychopathology may also vary as a function of racial/ethnic identity due to cultural differences. Specifically, investigators caution against viewing PWB as a desirable or beneficial mental health outcome within individuals belonging to a more collectivistic identity (e.g., Christopher, 1999), as the principles underlying PWB appear antithetical to those identities (mastery of one’s environment versus achieving harmony with one’s environment; e.g., Harding et al., 2017; Joshanloo, 2013). As individuals belonging to certain racial/ethnic identities in the United States (e.g., those identifying as Black or Latinx) may have a more collectivistic orientation (Markus & Kitayama, 1991), PWB and psychopathology may be more closely related in White adolescents, for whom individualistic qualities are reinforced. Thus, the current study additionally aims to examine differences in relations between PWB and psychopathology as they relate to adolescent identity.

### The present investigation

The current investigation aims to understand the between- and within-person relations between two important constructs of mental health, PWB and psychopathology (depressive symptoms and externalizing behaviors), within adolescence. Data from a short-term (1 year), three-wave study is examined within a longitudinal, random intercept cross-lagged panel analytic approach to determine: if the cross-sectional and longitudinal relations between psychopathology and wellbeing are linear or curvilinear (Aim 1), the magnitudes of the cross-sectional relations between different forms of psychopathology and wellbeing (Aim 2), the directionalities and magnitudes of the longitudinal relations between psychopathology and wellbeing (i.e., cross-lagged paths) (Aim 3), and the impact of gender and racial/ethnic identity on the paths between psychopathology and wellbeing (Aim 4). It was hypothesized that, in line with previous literature highlighting discrepancies in the relation between PWB and psychopathology (i.e., Ansary & Luthar, 2009; Salmivalli et al., 2000), a quadratic approach would be incrementally valid to a linear conceptualization (Aim 1), such that moderate levels of internalizing distress and externalizing behaviors related to the highest levels of PWB. With regard to cross-sectional relations (Aim 2), it was hypothesized that both internalizing distress and externalizing behaviors would have a small-moderate negative effect size similar to what has been previously found in the adolescent literature for internalizing distress and PWB (e.g., Ford et al., 2013). In light of prior work demonstrating that psychopathology (i.e., primarily indicators of internalizing distress) uniquely predicts PWB in adults, it was hypothesized that psychopathology would prospectively predict PWB (Aim 3) as opposed to PWB predicting psychopathology. Finally, we predicted that internalizing distress and PWB would be more closely associated in girls relative to boys, while all other potential demographic differences were probed in an exploratory fashion (Aim 4).

## Methods

### Participants

The baseline sample for the current study stems from an overarching study examining mental health outcomes among adversity-exposed youth. Specifically, youth lived either in a large, urban setting in southeast Texas (*N* = 507), or were connected with a juvenile justice system (JJS) diversion program in a small city in Illinois (*N* = 77). Youth were connected to the diversion program in various ways including being referred by police for a low-level infraction, referred by the school for behavioral problems (e.g., truancy), or through community events targeting families who are potentially at-risk for involvement with the JJS. Demographically, the Texas and Illinois samples were similar, with the only significant differences being across gender and race/ethnicity. Specifically, the sample from Texas more commonly identified as Hispanic (48% relative to 8%), χ^2^(1) = 42.05; *p* < 0.05, and the sample in Illinois tended to identify more as White (50% relative to 37%), χ^2^(2) = 6.39; *p* < 0.05. Finally, the only other differences were that the Illinois sample was lower on PWB at baseline, *t* (551) = −4.02; *p* < 0.01, and violent behavior at Wave 3 (t(344.06) = −3.65; *p* < 0.01). These differences between recruitment sources reflected demographic compositions of the sites, and did not exhibit consistent, systematic differences in the constructs being examined. As such, and in line with large public domain datasets for vulnerable populations, such as the Longitudinal Studies on Child Abuse and Neglect, which intentionally recruit and combine samples from geographically different sites to ensure findings are not contributed to specific subsamples and demonstrate variability in outcomes of interest (Runyan et al., 2014), we perform analyses by including participants from both sites. Of the 584 participants in the overall study, 553 participants completed traditional mental health self-report forms (i.e., depressive and violent-delinquent behaviors) and PWB at baseline. Approximately 71% (*N* = 391) completed measures at Wave 2, while approximately 66% (*N* = 364) completed measures at Wave 3. Within the analytic sample, the mean age was 14.97 years (SD = 1.21), and 51.2% of the sample identified as male at baseline. 40.7% of participants identified as Hispanic (225 individuals), 38.5% identified as White (213 individuals), and 35.6% identified as Black (197 individuals). Percentages total over 100, as participants were able to identify with multiple categories.

### Procedure

Youth in the southeast Texas sample were introduced to the study within school settings, and if interested, youth were provided with parental consent forms. Youth located in the midwestern United States sample were referred to the study by a case manager at a juvenile diversion program or were introduced to the study at a community event sponsored in part by the juvenile diversion program. At the event, if youth were alone, they were provided with consent-to-contact forms to sign so that the study team could contact caregivers. Subsequently, caregivers accompanied adolescents to the baseline session to sign the consent form. After this stage, procedures were identical across sites. Subsequent to providing assent, adolescents participated in a 45-minute baseline survey that assessed demographic characteristics, life events, and mental health. Approximately 6 months *(M* = 5.44) after the baseline visit (beginning in March 2020), adolescents completed a second survey (Wave 2) and a third survey (Wave 3) approximately six months later (*M* = 6.13) (beginning in October 2020). The survey protocol was identical across waves. The institutional review board at the last author’s institution approved this study.

### Measures

#### Depressive symptoms (PHQ, waves 1 – 3)

Adolescent depression was assessed using the nine-item Patient Health Questionnaire (PHQ-9; Kroenke et al., 2001). Example items included, “Trouble falling asleep, staying asleep, or sleeping too much” and “Trouble concentrating on things like school work, reading, or watching TV.” Reflecting on the previous two weeks, adolescents indicated the frequency of symptoms across response options, including not at all (0), several days (1), more than half the days (2), and nearly every day (3). Previous empirical literature demonstrates the reliable and valid use of this measure among adolescents (Richardson et al., 2010). Within the current study, the alpha reliability for this measure was acceptable at all timepoints of the study (α_W1_ = 0.92, α_W2_ = 0.90, α_W3_ = 0.93).

#### Violent-delinquent behaviors (VDS, waves 1 – 3)

To understand adolescent engagement in violent-delinquent behaviors, youth answered the eight-item Violence Delinquency Scale (VDS; Broidy et al., 2003). Reflecting on the past 6 months, youth answered items regarding activities, including “I participated in fighting with weapons” and “I participated in carrying a weapon that is not a gun”, with response options including never, once or twice, a few times, many times, and not in the past 6 months but in my lifetime. The variable was coded as such: never/not in the past 6 months but in my lifetime (1), once or twice (2), a few times (3), and many times (4). Past empirical literature has demonstrated the reliable and valid use of the VDS (Gold et al., 2011). Within the current study, the alpha reliability for this measure was acceptable at all timepoints of the study (α_W1_ = 0.84, α_W2_ = 0.84, α_W3_ = 0.86).

#### Psychological wellbeing (PWB, waves 1 – 3)

A version of the PWB Posttraumatic Change Questionnaire (PWB-PTCQ; Joseph et al., 2012) was utilized within the current study. The PWB-PTCQ is an 18-item self-report that queries adolescents regarding facets of PWB within the past 6 months. Response options range from 1 (not at all) to 5 (very much so). Unlike the original study, the present questionnaire did not tie answers to a specific event, which allowed for measurement of PWB in a manner that aligns with Ryff’s (1989) original conceptualization. Example items include “I have confidence in my opinions” and “I am able to cope with what life throws at me.” Past research found this measure reliably captured a unidimensional conceptualization of PWB in a diverse sample of adolescents over time (Thakur et al., 2024). Within the current study, the alpha reliability for this measure was acceptable at all timepoints of the study (α_W1_ = 0.97, α_W2_ = 0.98, α_W3_ = 0.98).

#### Demographics (Wave 1)

Three key demographics were retained within the dataset (i.e., age, gender, and race). Age was coded as a categorical variable (12 years old, 13 years old, 14 years old, 15 years old, 16 years old, 17 years old, 18 years old). Gender was a binary variable (Male, Female), while race was dichotomized into three non-mutually exclusive categories to allow for those identifying as multi-racial to be captured in all possible racial categories (Hispanic or not Hispanic, White or not White, Black or not Black).

### Data analytic plan

Missing data was examined via the Little’s Missing Completely at Random (MCAR) test. If significant, indicating data were not MCAR, supplemental analyses tested whether the data were missing at random (MAR). Specifically, chi-square and one-way ANOVA tests examined whether those who had missed a follow-up differed from those who had complete data on all study variables. Significant differences would signify data is not missing at random. Across analyses, expectation maximization methods were used for imputation.

Random intercept cross-lagged panel models (RI-CLPMs) were used to explore the aims of the current study. RI-CLPMs allow for modeling of trait-like stability in a time invariant manner through random intercepts (i.e., between person differences), as well as individual fluctuations around one’s expected score (i.e., within person differences) (Hamaker et al., 2015). Other modeling of longitudinal data (e.g., traditional cross-lagged panel models) does not parse between within-person and between-person effects, running the risk of conflating these two sources of information within the interpretation of results (Hamaker et al., 2015). Within the RI-CLPMs tested, mean scale scores were entered into a multivariate model to examine the acceptability of a RI-CLPM model inclusive of PWB, depression, and violent-delinquent behavior. Once acceptable fit was established with mean scale scores, a series of models were run in which depression and violent-delinquent behavior scores were entered as polynomial terms, to capture possible quadratic relations between psychopathology and PWB (Aim 1). Three sets of quadratic models were run, including one in which both psychopathology terms were quadratic, and two in which one psychopathology variable was quadratic. This allowed for an examination of the possibility that quadratic effects provided an incremental better fit for depression or violent-delinquent behavior separately. Linear effects would suggest that, across cross-lagged and covariance paths, each standard deviation unit variation in PWB, for example, is associated with a standard deviation variation in depressive symptoms that is equal to that path coefficient for all values of PWB (e.g., Kock & Gaskins, 2016). However, within a quadratic approach, this consistent relation in standard deviation variation is untenable, and instead requires a visual inspection of the relation, with a negative path coefficient suggesting an inverted-U relation between the constructs (e.g. see Ahrholdt et al., 2019). Finally, after determining whether linearity or non-linearity was preferred for the RI-CLPM, paths were examined to understand the magnitude and significance of the correlations at the between-person and within-person levels (Aim 2), and of the cross-lagged paths at the within-person level (Aim 3). Next, the moderating role of gender and race/ethnicity on relations was examined by constraining parameters across groups (Aim 4). This was accomplished by examining models in which paths were freely estimated (i.e., baseline model) to one in which paths were constrained to be equal across groups (i.e., stationary model). If no substantial difference in fit was observed by constraining parameters to be equal, then moderation was not supported (i.e., change in CFI ≤ 0.01).

Given the fewer degrees of freedom associated with random-intercept cross-lagged panels, investigators have cautioned against the use of RMSEA and x^2^/df for model fit, as these indicators unfairly penalize models with smaller degrees of freedom (Shi et al., 2022). It is instead recommended that a multi-indicator approach with comparative fit index (CFI) and standardized root mean squared residual (SRMR) be utilized to determine fit, which is in line with Hu and Bentler’s (1999) recommendation to use SRMR in combination with at least one other indicator. Thus, acceptable model fit was achieved if CFI ≥ .95 and SRMR ⩽ 0.08 (Schreiber et al., 2006). To compare models that are unnested, the Akaike Information Criteria (AIC) and Bayesian Information Criteria (BIC) were used, with a change in AIC and BIC ≥10 akin to model improvement (Burnham & Anderson, 2004). As χ^2^ difference tests exhibit sample size and non-normality sensitivity (Kline, 2016), a cutoff of ΔCFI ≤ .01 was used to assessinvariance and significant differences between nested models (Cheung & Rensvold, 2002). All ΔCFIs reported in the manuscript represent the absolute value of change.

## Results

### Preliminary analysis

Within the dataset, MCAR analyses were significant χ^2^ (43) = 76.422; *p* <0.01. In response, we explored if data was MAR by testing if those who completed all three waves differed from those with missing data across any baseline variables. Pearson chi-square tests indicated a significant association between gender and missingness at follow-up (χ(2) = 11.72, *p* = 0.003), such that males were more likely to miss a follow-up. No other differences were found across study variables, *p* > .05. Given that most of our study variables were not impacted by missing data, we used expectation maximization to impute missing data prior to conducting our analyses. Descriptive statistics before and after imputation are provided in Table 1 and bivariate correlations are displayed in Table 2.

As for our null models for each psychological construct, intraclass correlation coefficients examining between- and within-person variance for each outcome indicated that 62% of the variance in PWB (ICC = 0.38), 35% of the variance in depressive symptoms (ICC = 0.65), and 64% of the variance in violent-delinquent behaviors (ICC = 0.36) could be attributed to within-person differences. These findings supported the partitioning of between- and within-person variance within the random-intercept cross-lagged approach.

### Model building

Following the steps outlined by Mulder and Hamaker (2021), fit of an unconstrained RI-CLPM was examined. Across linear and quadratic models, fit was acceptable (CFI_linear_ = 0.96, SRMR_linear_ = 0.025; CFI_quadratic_ = 0.97, SRMR_quadratic_ = 0.021). In terms of model assumptions, across the linear and quadratic models, tenability of the constraints on means and covariances was disproven (Change in CFI_linearmeans_ = 0.26, Change in CFI_quadraticmeans_ = 0.30, Change in CFI_linearcovariances_ = 0.038, Change in CFI_quadraticcovariances_ = 0.046), such that the unconstrained baseline model fit significantly better. In contrast, equality of paths was upheld across linear and quadratic models (Change in CFI_linearpaths_ = 0.016, Change in CFI_quadraticpaths_ = 0.027). More specifically, though fit was significantly different between the baseline and constrained models, the constrained model provided significantly better fit for the linear and quadratic frameworks. This pattern of equality constraints maintained within models in which a single psychopathology measure was quadratic. As such, the baseline model across linear and quadratic models consisted of constrained autoregressive and cross-lagged paths across time.

#### Model selection (aim 1)

To understand whether it was best to proceed with linear or quadratic conceptualizations of psychopathology, AIC and BIC values were compared across models. This comparison indicated that the linear formulation was preferred (AIC: 7281.16, BIC: 7462.40) to all potential quadratic formulations (AIC_PHQandVDSquadratic_ = 16,454.12, BIC_PHQandVDSquadratic_ = 16,635.37; AIC_PHQquadratic_ = 12,215.21, BIC_PHQquadratic_ = 12,396.46; AIC_VDSquadratic_ = 11,522.53, BIC_VDSquadratic_ = 11,703.78). Thus, it was concluded that the relation between PWB and psychopathology was best defined as linear and this model was retained for further analyses. The CFI for the linear constrained model was 0.98, and the SRMR was 0.041.

#### RI-CLPM significant paths (aims 2 and 3)

Within the linear model, several significant findings emerged. To facilitate interpretation, standardized coefficients are presented here and in Table 3. First, with regard to correlations between our random intercepts, the correlation between depression and PWB (*b*_PHQ_ = −0.25, SE = 0.11, *p* = 0.021), as well as violent-delinquent behavior and PWB (*b*_VDS_ = −0.39, SE = 0.15, *p* = 0.011) was negative. Thus, both depression and violent-delinquent behavior exhibited a small, negative, cross-sectional, between-person relation with PWB. With regard to within-person cross-sectional correlations, only two significant associations emerged. Depressive symptoms negatively related to PWB at baseline (*b*_PWB_ = −0.34, SE = 0.061, *p* < 0.01) and violent-delinquent behavior was positively related to PWB at Wave 3 (*b* = 0.21, SE = 0.080, *p* < 0.01), suggesting those who exhibited heightened violent-delinquent behavior also concurrently experienced elevated PWB. The effect size of depressive symptoms and PWB at Wave 2 was similar to that at baseline (albeit, not statistically significant), while the cross-sectional relation between PWB and violent-delinquent behavior was inconsistent across waves (see Table 3).

With regard to the prospective, cross-lagged paths (Aim 3), we did not identify any prospective relation between depressive symptoms and PWB, *ps* > .05. However, violent-delinquent behaviors at time t-1 significantly predicted PWB at time t at all waves (*b*_PWBW2_ = 0.21, SE_PWBW2_ = 0.076, *p* < 0.01; *b*_PWBW3_ = 0.14, SE_PWBW2_ = 0.051, *p* < 0.01), albeit in a positive direction. Similar to the cross-sectional relation at Wave 3, these findings suggested that upward fluctuations in violent-delinquent symptoms prospectively predicted upward fluctuations in PWB. We did not find any examples of PWB prospectively predicting patterns of psychopathology, *p* > .05.

### Moderation (aim 4)

To test for moderating effects, we first examined baseline models in which autoregressive and cross-lagged paths were constrained to be equal across time and the correlations between random intercepts were constrained across groups (i.e., between violent-delinquent behaviors and depressive symptoms, between depressive symptoms and PWB, and between violent-delinquent behaviors and PWB) to a model in which correlations between random intercepts were freely estimated. Across gender and dummy-coded racial/ethnic variables, this comparison indicated the absence of moderating effects (Change in CFI_gender_: 0.004, Change in CFI_White_: 0.012, Change in CFI_Black_: 0.01, Change in CFI_Hispanic_: 0.001). Given the lack of moderation for the correlation between random intercepts, we subsequently tested the moderating effect of autoregressive and cross-lagged paths by comparing a model in which random intercepts were held the same across groups and autoregressive and cross-lagged paths were held the same across time to a model that additionally constrained autoregressive and cross-lagged paths to be the same across groups. This comparison also demonstrated the absence of moderating effects (Change in CFI_gender_: 0.011, Change in FI_White_: 0.005, Change in CFI_Black_: 0.006, Change in CFI_Hispanic_: 0.002). Finally, we examined moderation for cross-sectional correlations between constructs. To test this, we compared a baseline model in which random intercepts were held the same across groups, cross-lagged/autoregressive paths were held the same across time and groups, and cross-sectional correlations were held the same across groups to a model in which cross-sectional correlations were freely estimated across groups. This comparison also demonstrated no moderation for gender and race/ethnicity (Change in CFI_gender_: 0.013, Change in CFI_White_: 0.001, Change in CFI_Black_: 0.01, Change in CFI_Hispanic_: 0.01). Overall, these findings suggest that the pathways outlined in Table 3 generalized to the entire sample.

## Discussion

The current study examined the relation between adolescent PWB and two forms of psychopathology (i.e., depressive symptoms and violent-delinquent behaviors) within a random intercept cross-lagged panel approach. Between-person findings indicated that adolescents higher on psychopathology consistently exhibit lower PWB than their peers. Similarly, the within-person relations between depressive symptoms and PWB exhibited a small-medium, negative effect. These findings aligned with hypotheses, past research with adults (Keyes, 2005), and extant theory, which posits negative and positive mental health states act antagonistically (Fredrickson et al., 2008). Alternatively, contrary to hypotheses, we found no predictive relation between depressive symptoms and PWB and, unexpectedly, found a positive, predictive relation between violent behavior and future PWB. Below, we contextualize these findings, and others, in the extant adult literature on positive and negative mental health (e.g., Kraiss et al., 2022), and elaborate on how these findings inform our understanding of adolescent mental health.

Empirical studies examining the relation between PWB and distress assume a linear relation between the two constructs (Lamers et al., 2015). Contrary to hypotheses, results from the current study support this assumption suggesting that increases or decreases in PWB are associated with a consistent change in psychopathology. From an applied perspective, this may suggest that current clinical screening and assessment programs focused on distress may be indexing holistic well-being as well. However, it may be premature to conclude that screening and assessment protocols solely rely on the use of distress indicators to capture holistic mental health. The effect size of the relations between distress and PWB suggests that though there is some overlap in construct content, they represent distinct entities (small to medium versus large effect size). In fact, previous research demonstrates meaningful statistical inferences when utilizing both indices for differentiation of mental health profiles within adolescence (Cohen et al., 2021). To best capture the integration of both indices within applied settings, it may be additionally necessary to establish clinically meaningful thresholds across both constructs, which would allow for interpretation of linear changes in measures and guide clinical decision-making that is patient-centered (e.g., minimally important differences; Jaeschke et al., 1989).

To date, few studies have disentangled between and within-person effects when examining PWB and internalizing symptoms. Kraiss and colleagues (2022) found that internalizing distress and PWB were negatively, cross-sectionally associated in a sample of emerging adults across between and within-person associations. Joshanloo and Blasco-Belled (2023) found significant, negative between-person correlations and cross-lagged effects between depressive symptoms and PWB in a sample of older adults. The present study’s findings are largely congruent with these studies, with both between and within-levels of depression and PWB exhibiting a negative, small-moderate association. That internalizing distress and PWB showed relatively similar relations in adults and adolescents suggests developmental continuity between positive and negative indices of mental health. Indeed, past empirical work demonstrates continuity in internalizing distress from adolescence to adulthood (Essau et al., 2014; Gutman & Sameroff, 2004), with aspects of PWB (i.e., less perceived social support; Stice et al., 2011; Strine et al., 2009) linked to depressive symptoms during these periods as well. Continuity in these presentations has been hypothesized to be maintained via several mechanisms, including cognitive factors that span adolescence through adulthood (e.g., depression; Hankin & Abela, 2005). As such, it is possible that cognitive mechanisms hypothesized to contribute to the link between psychopathology and PWB are maintaining this consistent relation. As an example, positive appraisal is linked to fewer depressive symptoms (Garnefski et al., 2002), as well as PWB (Puente-Martínez et al., 2018), which may make it a promising integrated factor for positive and negative mental health outcomes across adults and adolescents. Collectively, this would suggest that protocols, such as Well-Being Therapy (Fava, 1999), that leverage the negative relation between PWB and distress, and which target shared cognitive pathways, are well positioned to facilitate holistic mental health in a developmentally appropriate manner.

It is notable, however, that within-person findings between depressive symptoms and PWB were not consistently significant in the current study. Comparisons between the current study and prior research examining within- and between-person relations exhibit important differences. For example, Kraiss and colleagues (2022) conceptualized within-person relations using daily ecological momentary assessments over the course of two weeks. The measures within the current study were administered 6 months apart, which may suggest that PWB and depression may impact each other during shorter intervals. As such, detection of significant signals may occur with more proximal follow-up points. However, it is also possible that with the accumulation of more follow-up points (momentary assessments for 14 days; Kraiss et al., 2022; 8 intervals spanning two years; Joshanloo & Blasco-Belled, 2023), in contrast to the three waves of data in the current study, consistent significant findings would be better captured. Significant findings with shorter, more frequent time intervals would suggest that though depressive and PWB tend to negatively relate to each other at the within-person level, current long-term, retrospective based methods fail to capture the real-world dynamics of these constructs.

The proposed study also extended prior work on PWB and psychopathology by finding evidence of significant between and within-person effects for externalizing behaviors. At the between-person level, we found that violent behavior was negatively associated with PWB. This was consistent with our hypothesis and consistent with research that demonstrates that externalizing tendencies may lay the foundation for decreased PWB (poor interpersonal relationships; Sharp et al., 2011). Alternatively, our within-person findings suggested prospective increases in PWB for those exhibiting externalizing behaviors. Although not hypothesized, prior research conducted among adolescents suggests a protective, even positive, role of externalizing behaviors during this developmental period (Panayiotou & Humphrey, 2018). These findings are in line with theories that suggest delinquency arises in response to situations in which eudaimonic wellbeing, specifically positive relations with others, autonomy and environmental mastery (Self-Determination Theory; Deci & Ryan, 2012), appears unattainable through prosocial methods (Ward & Stewart, 2003). As an example of this phenomenon, focus group work demonstrated that adolescents aged 13–17 indicated that delinquency is exhibited when authority figures are perceived to be ineffective, thus motivating adolescents to rely on externalizing behaviors to resolve conflict (Shetgiri et al., 2015). It is also possible that there is a context specific effect observed within the current study, with data collection for follow-up periods overlapping with the COVID-19 pandemic. The violent-delinquent measure used within the current study captures physical aggression but also maps onto behaviors that imply an interpersonal context (e.g., participated in fist fighting, participated in throwing things at other people). Given that the pandemic led to unprecedented social isolation for adolescents (e.g., Cingel et al., 2022), it is possible that those reporting elevated aggressive and violent behaviors were defying social distancing policies for immediate social interaction gratification. These increases in opportunities to engage with others, although in an aggressive manner, may have led to increases in PWB. In other words, within the context of the COVID-19 pandemic, the violent, delinquent subscale may have acted as an indicator of interpersonal engagement rather than pure externalizing behaviors. In summary, our results suggest that those with elevated externalizing behaviors may currently experience lower levels of PWB relative to their peers, however, may experience, relative to themselves, higher future PWB.

The phenomenon of contrasting relations (i.e., positive vs. negative) across different levels of analysis (i.e., within vs. between) is referred to as the Simpson’s Paradox (Blyth, 1972) and exists for other psychological phenomena. For instance, at the between-person level, adolescents who endorse higher perceptions of privacy invasion by parents also report higher secrecy behaviors. However, at the within-person level, increased adolescent secrecy behaviors prospectively predict decreased parental privacy invasion behaviors (Dietvorst et al., 2018). For our study, it may be that displaying externalizing behaviors leads to more environmental stressors (e.g., punishment) concurrently, leading to lower levels of PWB for these youth relative to their peers. However, prospectively, once the punitive consequences fade, acting aggressively may be positively reinforced during adolescence via admiration from peers or reinforcing through engagement in environmental mastery (e.g., maintaining respect among peers, perception of authority figures as ineffective; Shetgiri et al., 2015). Critiques of the Simpson’s Paradox suppose that its presence signals a potential moderating or quadratic relation (Kock & Gaskins, 2016). However, as the current study explicitly explored potential moderating *and* quadratic effects for the relation between PWB and distress, it is likely that the current findings do not reflect the methodological error and is a finding that warrants further attention.

This Simpson’s Paradox has important implications for intervening with externalizing behaviors. Predominant interventions for externalizing behaviors (i.e., Defiant Teens; Barkley & Robin, 2014) identify reduction in parent-child conflict as a primary therapeutic target and subscribe to social learning models (i.e., coercion theory; Patterson, 2016), which assert that externalizing behaviors are learned and reinforced through familial interactions. As such, techniques employed in intervention focus on the shaping of parental behavior, including consistent parenting practices, delivery of positive attention, and reinforcement of prosocial behavior (Barkley & Robin, 2014). These approaches map onto the between-person findings within the current study, which suggest that disrupting tendencies to engage in delinquent behaviors may be associated with higher wellbeing tendencies. However, it is possible that predominant intervention approaches miss other motivations for engagement in externalizing behavior, such as opportunities to connect with peers who provide meaningful sources of community (Barnert et al., 2015). Within this context, focusing on the parent-child relationship will miss the main motivation for the delinquent behavior. Instead, interventions should focus on providing other opportunities for youth to socially connect and on demonstrating prosocial ways to exercise one’s autonomy (Larson et al., 2007). Without consideration for the nomological network surrounding externalizing behaviors and positive wellbeing, clinicians run the risk of focusing too narrowly on reducing externalizing behaviors, without maximizing solutions that simultaneously manage delinquent behaviors while cultivating PWB at the individual level.

Given the importance of understanding demographic characteristics on mental health phenomena (e.g., Nolen-Hoeksema & Girgus, 1994), this study additionally examined considerations that the relations between PWB and psychopathology may differ by gender and racial identities. Ultimately, results indicated a lack of moderation for demographic characteristics (i.e., race, gender) for within-person and between-person relations. It is important to note, however, that identity was measured simplistically with the use of common demographic responses (i.e., Male or Female). It is possible that identity-related differences in the holistic mental health framework are elicited by focusing on experiences or processes (e.g., masculinity/femininity; Rogers et al., 2017), rather than relying on identity categories. In addition, the time period and methodology (i.e., 1 year timespan) associated with data collection did not allow for investigation into changes in the relations between PWB and psychological distress across adolescent development. More specifically, examination of within- and between-person relations for individuals as they progress through adolescence (i.e., early to middle to late stages) would provide insight into normative trait fluctuations at the population level and state fluctuations at the individual level. Insight into normative development would subsequently better inform establishment of clinically meaningful thresholds for these relations and allow for better distinguishing between context-dependent and development-dependent impacts.

Although the current study possesses several methodological strengths, including the use of a racially/ethnically-diverse United States-based population, several limitations can help to inform future directions. As is common with longitudinal data, the current study experienced attrition, but uniquely overlapped with an emergent public health crisis. As such replication of this study with other longitudinal samples will help to strengthen generalizability. The present study relied on self-report for measures. Although there are benefits to utilizing adolescents as their own informants (Tein et al., 1994), the use of multiple informants represents an important next direction. It is possible that differences between an adolescent’s reported relations between psychological distress and PWB, and another informant’s report, would provide insight into how psychopathology and PWB impact each other across different settings (e.g., De Los Reyes et al., 2013). Finally, it is important to consider that current methods for measuring PWB and distress may benefit from continued examination. As an example, other measures of wellbeing employed within adolescence include age and developmental specific behaviors (Self-Perception Profile for Children (Harter, 1985) versus Self-Perception for Adolescents (Harter, 2012)), with the intention of more accurately capturing constructs across these transition points. It is possible that PWB measurement may similarly benefit from understanding the constellation of behaviors that best characterize PWB within early, middle and late adolescence. In addition, current measures are limited in their ability to capture holistic mental health effects in non-physical contexts. As adolescents increasingly incorporate non-physical interactions into their daily lives via an online presence (e.g., Anderson et al., 2023), the translation of the holistic mental health framework to this context will be important to explore. In fact, investigators theorize that online behaviors represent a method for coping with distress (Theory of Compensatory Internet Use; Kardefelt-Winther, 2014) and reflect engagement with PWB-related behaviors (Ross & Tolan, 2021), which would suggest there may be opportunities to beneficially leverage the holistic mental health framework outside traditional approaches. As such, characterization of PWB and distress in non-physical spaces, with considerations for important age and developmental behavioral manifestations, may not only better capture the holistic mental health framework, but also represent the next frontier for culturally and developmentally appropriate assessment and intervention efforts during the adolescent epoch.

## Figures and Tables

**Table 1.** Means and standard deviations of sum score before and after imputation

Sum Variable (N_unimputed_, N_imputed_)	Unimputed mean (Imputed mean)	Unimputed standard deviation (imputed standard deviation)

PWB Wave 1 (553, 553)	67.094 (67.094)	17.84 (17.84)
PWB Wave 2 (387, 553)	58.91 (59.64)	19.92 (17.41)
PWB Wave 3 (362, 553)	55.27 (55.30)	20.23 (17.17)
PHQ Wave 1 (553, 553)	6.59 (6.62)	6.66 (6.66)
PHQ Wave 2 (391, 553)	7.06 (6.82)	6.36 (5.74)
PHQ Wave 3 (364, 553)	7.37 (6.93)	7.15 (6.24)
VDS Wave 1 (553, 553)	9.44 (9.44)	3.052 (3.052)
VDS Wave 2 (389, 553)	8.85 (8.90)	2.40 (2.12)
VDS Wave 3 (364, 553)	8.70 (8.69)	2.30 (1.95)

*Note*. PWB = Psychological Wellbeing Posttraumatic Change Questionnaire (Joseph et al., 2012), Patient Health Questionnaire (Kroenke et al., 2001), Violence Delinquency Scale (Broidy et al., 2003). PHQ scored on a 0 (not at all) to 3 (nearly every day) scale. PWB scored on a 1 (not at all) to 5 (very much so) scale. VDS scored on a 1 (never/not in the past 6 months but in my lifetime) to 4 (many times) scale.

**Table 2.** Correlations between imputed sum score variables

	PHQ W1	PHQ W2	PHQ W3	PWB W1	PWB W2	PWB W3	VDS W1	VDS W2	VDS W3

PHQ W1	1	.640**	.613**	−.372**	−.269**	−.072	.269**	.107*	.223**
PHQ W2	.640**	1	.722**	−.237**	−.197**	−.070	.136**	.237**	.238**
PHQ W3	.613**	.722**	1	−.222**	−.172**	−.062	.058	.110**	.236**
PWB W1	−.372**	−.237**	−.222**	1	.436**	.375**	−.157**	−.151**	−.145**
PWB W2	−.269**	−.197**	−.172**	.436**	1	.523**	−.001	−.051	−.089*
PWB W3	−.072	−.070	−.062	.375**	.523**	1	−.072	−.086*	−.026
VDS W1	.269**	.136**	.058	−.157**	−.001	−.072	1	.383**	.380**
VDS W2	.107*	.237**	.110**	−.151**	−.051	−.086*	.383**	1	.437**
VDS W3	.223**	.238**	.236**	−.145**	−.089*	−.026	.380**	.437**	1

*Note*. PHQ = Patient Health Questionnaire (Kroenke et al., 2001), PWB = Psychological Wellbeing Posttraumatic Change Questionnaire (Joseph et al., 2012), VDS = Violence Delinquency Scale (Broidy et al., 2003). W1=Wave 1, W2=Wave 2, W3=Wave 3. PHQ scored on a 0 (not at all) to 3 (nearly every day) scale. PWB scored on a 1 (not at all) to 5 (very much so) scale. VDS scored on a 1 (never/not in the past 6 months but in my lifetime) to 4 (many times) scale.

** Correlation is significant at the 0.01 level (2-tailed).

* Correlation is significant at the 0.05 level (2-tailed).

**Table 3.** Summary of findings from linear random intercept cross-lagged panel model with fixed autoregressive and cross-lagged paths

Path	Standardized coefficient	SE	*p*-value

Correlation between random intercepts (PWB and VDS)	−0.39	0.15	<0.05
Correlation between random intercepts (PWB and PHQ)	−0.25	0.11	<0.05
Correlation between random intercepts (VDS and PHQ)	0.29	0.06	<0.01
VDS 1 -> PWB 2	0.21	0.08	<0.01
VDS 2 -> PWB 3	0.14	0.05	<0.01
VDS 1 -> PHQ 2	0.14	0.09	0.11
VDS 2 -> PHQ 3	0.06	0.04	0.12
PHQ 1 -> PWB 2	−0.24	0.14	0.09
PHQ 2 -> PWB 3	−0.14	0.07	0.06
PHQ 1 -> VDS 2	−0.04	0.08	0.60
PHQ 2 -> VDS 3	−0.03	0.05	0.60
PWB 1 -> VDS 2	0.03	0.07	0.69
PWB 2 -> VDS 3	0.03	0.08	0.71
PWB 1 -> PHQ 2	−0.17	0.17	0.31
PWB 2 -> PHQ 3	−0.12	0.10	0.23
VDS 1 -> VDS 2	0.10	0.10	0.32
VDS 2 -> VDS 3	0.07	0.08	0.35
PWB 1 -> PWB 2	0.17	0.13	0.21
PWB 2 -> PWB 3	0.17	0.15	0.27
PHQ 1 -> PHQ 2	−0.22	0.17	0.20
PHQ 2 -> PHQ 3	−0.08	0.05	0.07
Correlation between VDS 1 and PWB 1	−0.07	0.08	0.38
Correlation between PHQ 1 and PWB 1	−0.34	0.06	<0.01
Correlation between VDS 1 and PHQ 1	0.30	0.06	<0.01
Correlation between VDS 2 and PWB 2	0.11	0.08	0.18
Correlation between PHQ 2 and PWB 2	−0.25	0.28	0.38
Correlation between VDS 2 and PHQ 2	0.31	0.11	<0.01
Correlation between VDS 3 and PWB 3	0.21	0.08	<0.01
Correlation between PHQ 3 and PWB 3	−0.02	0.08	0.84
Correlation between VDS 3 and PHQ 3	0.08	0.08	0.32

*Note*. PHQ = Patient Health Questionnaire (Kroenke et al., 2001), PWB = Psychological Wellbeing Posttraumatic Change Questionnaire (Joseph et al., 2012), VDS = Violence Delinquency Scale (Broidy et al., 2003). 1=Wave 1, 2=Wave 2, 3=Wave 3. PHQ scored on a 0 (not at all) to 3 (nearly every day) scale. PWB scored on a 1 (not at all) to 5 (very much so) scale. VDS scored on a 1 (never/not in the past 6 months but in my lifetime) to 4 (many times) scale.

## Data Availability

This data can be accessed at: https://doi.org/10.3886/ICPSR38324.v1
